# Stereoselective Synthesis of α-Amino-*C*-phosphinic Acids and Derivatives

**DOI:** 10.3390/molecules21091141

**Published:** 2016-08-29

**Authors:** José Luis Viveros-Ceballos, Mario Ordóñez, Francisco J. Sayago, Carlos Cativiela

**Affiliations:** 1 Secretaría Académica, Universidad Autónoma del Estado de Morelos, Av. Universidad 1001, 62209 Cuernavaca, Morelos, Mexico; jlvc@uaem.mx; 2 Centro de Investigaciones Químicas-IICBA, Universidad Autónoma del Estado de Morelos, Av. Universidad 1001, 62209 Cuernavaca, Morelos, Mexico; 3 Departamento de Química Orgánica, Universidad de Zaragoza-CSIC, ISQCH, 50009 Zaragoza, Spain; jsayago@unizar.es

**Keywords:** α-amino-*C*-phosphinic acids, α-amino-*C*-phosphinates, α-amino acids, stereoselective synthesis, biological activity

## Abstract

α-Amino-*C*-phosphinic acids and derivatives are an important group of compounds of synthetic and medicinal interest and particular attention has been dedicated to their stereoselective synthesis in recent years. Among these, phosphinic pseudopeptides have acquired pharmacological importance in influencing physiologic and pathologic processes, primarily acting as inhibitors for proteolytic enzymes where molecular stereochemistry has proven to be critical. This review summarizes the latest developments in the asymmetric synthesis of acyclic and phosphacyclic α-amino-*C*-phosphinic acids and derivatives, following in the first case an order according to the strategy used, whereas for cyclic compounds the nitrogen embedding in the heterocyclic core is considered. In addition selected examples of pharmacological implications of title compounds are also disclosed.

## 1. Introduction

**Figure 1:**
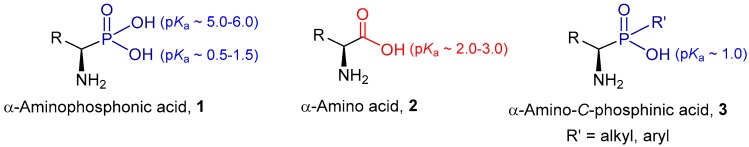
General structure for α-amino acids (**2**) and their phosphonic (**1**) and *C*-phosphinic (**3**) analogues.

Optically active α-aminoalkylphosphonic acids **1** are probably the most important analogues of the α-amino acids **2**, obtained by isosteric substitution of the planar and less bulky carboxylic acid (CO_2_H) by a tetrahedral phosphonic acid functionality (PO_3_H_2_). These classes of compounds are currently attracting great interest in organic and medicinal chemistry, as well as in agriculture, due to their important biological and pharmacological properties [1,2,3,4,5]. The great importance of this type of compounds has prompted organic chemists to report numerous procedures for their racemic or stereoselective synthesis [6,7,8,9,10,11]. On the other hand, the optically active α-amino-*C*-phosphinic acids **3** are also considered as analogues of α-amino acids **2** where now the carboxylic group (CO_2_H) is replaced by a tetrahedral and sterically more demanding phosphinic acid functionality (PO_2_HR′). As part of peptides, α-amino-*C*-phosphinic acids **3** are much closer analogues to natural α-amino acids **2** than their α-aminophosphonic counterparts **1**, due to their mono acidic character, and the higher stability of the P-C bond of **3** compared to the P-O bond of **1** (Figure 1) [12].

Phosphinic, phosphonic and carboxylic acids differ in several characteristics. The carboxylic group is planar at carbon while their phosphorus analogues are tetrahedral and considerably larger in size (the phosphorus atom has a much larger atomic radius than carbon). Phosphinic and phosphonic acids exist mainly in their tetracoordinate form, and they are considered as mono- and diacids, respectively. The phosphinic acids (p*K*_a_ ~ 1.0) are slightly stronger than the corresponding phosphonic acids (p*K*_a_ ~ 0.5–1.5) and both more acidic than the carboxylic acid equivalent (p*K*_a_ ~ 2.0–3.0); the former are mono acids and no evidence for the second acidity due to the P-H bond is reported, while for phosphonic acids, it is well-known that the second acidity is about 5 p*K* units higher than the first P-OH acidity. Similar to α-amino acids, the aminophosphonic and aminophosphinic acids have a zwitterionic form due to the internal hydrogen transfer between the P-OH and amino groups [13,14]. In addition, a characteristic feature of tetracoordinated phosphorus (III) compounds such as *H*-phosphonate and *H*-phosphinate derivatives, is the configurational stability of the phosphorus center at room temperature, and their tautomeric equilibria with trivalent species that occurs without epimerization at the phosphorus center. This is the basis for synthetic applications of these compounds as chiral precursors in several stereospecific transformations [15]. Although, different structures of phosphonic and phosphinic groups compared with the carboxylic function could a priori disrupt enzyme-ligand interactions, they frequently are recognized by enzymes or receptors as inhibitors or false substrates [16,17].

**Figure 2:**
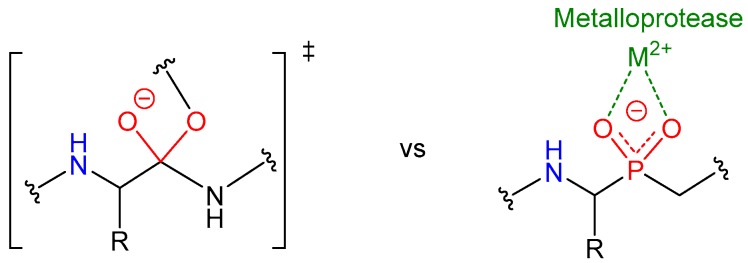
Similarity between the transition state of peptide hydrolysis and the α-amino-*C*-phosphinic motif. The additional interaction with the metal ion in the metalloproteases active site is included.

Phosphinopeptides containing *C*-terminal α-aminophosphinic acids have been prepared by similar methods to those used with their phosphonic analogues, for example the coupling of *N*-protected amino acids or their active esters with α-aminophosphinates [18], or with free α-aminophosphinic acids in organic or aqueous-organic media has been reported [19]. Additionally, the synthesis of phosphinopeptides via the Mannich-type condensation of *N*-Cbz protected alkanamides/peptide amides, aldehydes and aryldichlorophosphines, followed by hydrolysis, has also been reported [20]. Therefore, the α-amino-*C*-phosphinic acids are currently attracting interest in medicinal chemistry due to their relevance mainly as pseudopeptides, which have acquired pharmacological importance in influencing physiologic and pathologic processes, primarily acting as inhibitors for proteolytic enzymes. The success of these compounds is based on the resemblance of phosphinic acids to the sp^3^ transition state of the hydrolysis of peptide bonds. Considering that lengths of O-P and C-P bonds are significantly longer than the corresponding O-C and C-C bonds, the phosphinic fragment might be considered as an extended tetrahedral intermediate and thus can be treated similar to the transition state of the hydrolysis (Figure 2) [21]. Moreover, they can act as simple analogues of amino acids replacing the carboxylic group by the phosphinic moiety, as non-hydrolysable phosphate analogues or as chelating agents of metal ions present in the active site of the enzymes [22]. A number of excellent reviews on various aspects of their activity in natural systems have been published [16,17].

Simple pseudopeptides containing α-amino-*C*-phosphinate moieties replacing the scissile peptide bonds, rank among most potent inhibitors of metalloproteinases, where their tumorigenesis and invasion-related functions makes these enzymes molecular targets for the development of potential anticancer drugs [23]. The role of neutral aminopeptidase in the pathogenesis of hypertension provides an opportunity for regulating arterial blood pressure through its inhibition with this class of compounds [24]. Furthermore, these pseudopeptides appear to be excellent inhibitors to *Plasmodium falciparum* aminopeptidases, promising targets for a novel treatment of malaria [25,26]. α-Amino-*C*-phosphinic pseudopeptides have also revealed their potential activity in the regulation of matrix metalloproteinases (MMPs), whose overexpression or inadequate levels leads to pathological states such as osteoarthritis, rheumatoid arthritis and inflammation, but also it is associated with tumor growth, invasion and metastasis [27,28]. Additionally, is noteworthy that in these derivatives the stereochemistry affects their biological activity, hence the importance of the synthesis of these compounds in enantiomerically pure form [29,30].

**Scheme 1:**
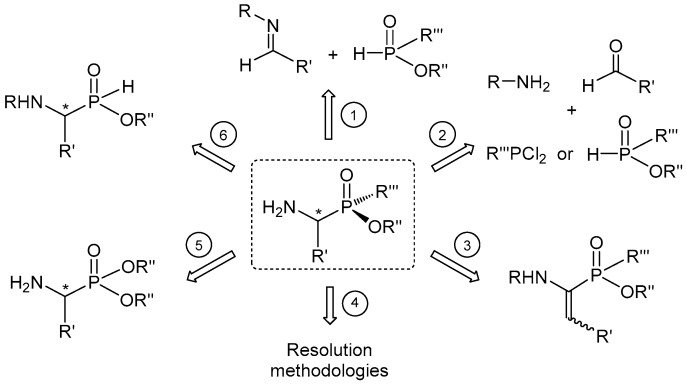
General overview of the synthetic strategies leading to α-amino-*C*-phosphinic acids.

In view of the broad biological applications of the optically active α-amino-*C*-phosphinic acids and their peptidic derivatives, in the last years the development of suitable synthetic methodologies for their preparation in optically pure form has been a topic of great interest in several research groups [31,32]. In this context, now we would like to report herein a summary of the stereoselective synthesis of α-amino-*C*-phosphinic acids and their derivatives covering the last 20 years. In this review article, the compounds have been classified in acyclic and phosphacyclic α-amino-*C*-phosphinic derivatives. In the first case an order is established according to the strategy used, in this way all procedures can be classified into C-P bond formation using a Pudovik or Kabachnik-Fields like reaction, C-H bond formation derived from catalytic asymmetric hydrogenation of α,β-dehydroaminophosphinates, resolution methodologies, preparation from chiral α-amino-phosphonates and also P-C bond formation from chiral α-amino-*H*-phosphinic derivatives is included (Scheme 1). Otherwise, in the second case involving the phosphacyclic derivatives, the classification will be based in the presence or lack of the nitrogen atom in the heterocycle.

## 2. Stereoselective Synthesis of Acyclic α-Amino-*C*-phosphinic Acids and Derivatives

### 2.1. Stereoselective C-P Bond Formation (Addition of Phosphorus Compounds to Imines)

A strategy widely used for the stereoselective synthesis of α-amino-*C*-phosphinic acids is the addition of a nucleophile HX (X = CN, PO_2_R, PO_2_RR′) to a preformed *N*-substituted chiral imine in the presence or absence of a chiral catalyst [33], leading to optically active α-amino carboxylic, α-aminophosphonic and α-aminophosphinic acids precursors, respectively. The extent of asymmetric induction at the C_α_ depends on the nature of the substituents (steric or hydrophobic interactions) and nucleophile–substrate interactions, which are favored in organocatalytic processes [34].

**Scheme 2:**
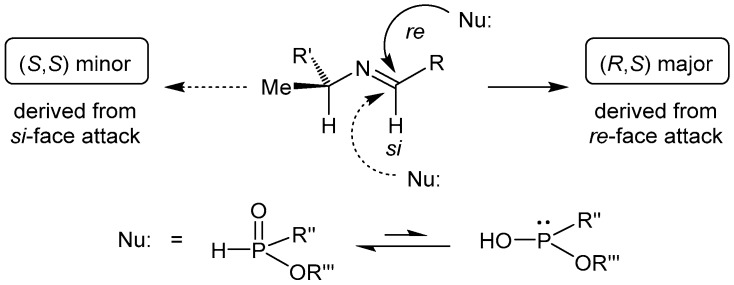
Model to explain the high diastereoselectivity in the nucleophilic addition to chiral imine compounds.

Indeed, the nucleophilic addition of phosphorus compounds (alkyl or aryl phosphinates) to imines, a Pudovik-like reaction [35], is an appropriate procedure for the preparation of α-amino-*C*-phosphinates. Generally, the high diastereoselective ratio can be explained on the basis of the most stable conformation of the Schiff bases, in which the C-H bond of the imine is eclipsed with the N–C–H fragment, as would be expected from the 1,3-allylic strain model [36,37], and the nucleophilic attack of the alkyl or aryl phosphinate takes place on the less hindered side (in the example below, R’ is bulkier than the methyl group) (Scheme 2).

**Scheme 3:**
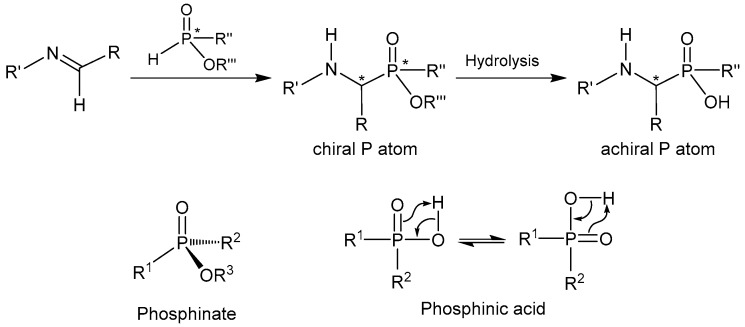
Structure of α-amino-*C*-phosphinates and α-amino-*C*-phosphinic acids.

Contrary to the phosphonate synthesis, the addition of *H*-phosphinates to imines results in the generation of two new stereogenic centers due to the attack of the chiral phosphinates at the prochiral C=N bond [38]; however, the phosphorus chirality is lost during the hydrolysis through the delocalization of P=O bond due to d-p π bond (Scheme 3) [28].

#### 2.1.1. Chiral Imine Compounds

**Scheme 4:**
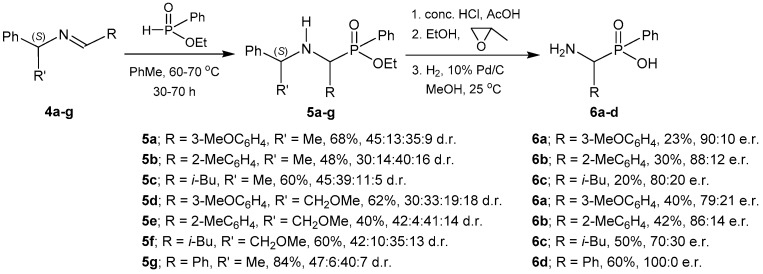
First enantioselective synthesis of α-amino-*C*-phosphinic acids **6a**–**d**.

Petneházy et al. [39] reported the first enantioselective synthesis of α-amino-*C*-phosphinic acids by the addition of phosphinates to chiral imines in the absence of a catalyst. In this regard, the (*S*)-α-methylbenzylamine and (*S*)-α-methoxymethylbenzylamine derived imines **4a**–**g** were reacted with ethyl phenylphosphinate in toluene at 70 °C, to obtain the α-amino-*C*-phosphinates **5a**–**g** in 40%–84% yield and moderate diastereoisomeric ratio. The hydrolysis of the phosphinate moiety in **5a**–**g** using concd. HCl or HBr/AcOH, followed by treatment with propylene oxide and hydrogenolysis using Pd/C in MeOH, provided the optically active α-amino-*C*-phosphinic acids **6a**–**d** in 20%–60% yield and with an enantiomeric ratio of good to excellent (Scheme 4).

**Scheme 5:**
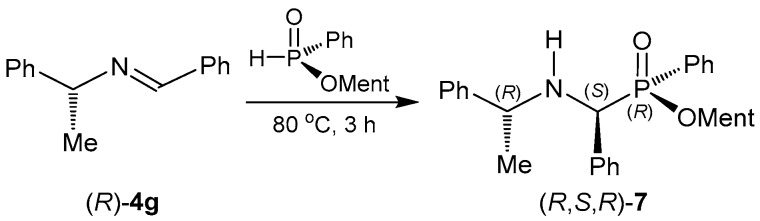
Synthesis of the *P*-chiral *O*-menthyl phosphinate (*R*,*S*,*R*)-**7**.

Recently, Zhao et al. [40] described the synthesis of a *P*-chiral compound, whose *R*_P_ chirality, was fully confirmed by X-ray analysis. In this context, the hydrophosphinylation of the *N*-(*R*)-α-methylbenzyl Schiff base (*R*)-**4g** with the (*R*_P_)-*O*-(−)-menthyl phenylphosphinate at 80 °C, gave after crystallization the optically pure *O*-menthyl phosphinate (*R*,*S*,*R*)-**7** (Scheme 5).

**Scheme 6:**
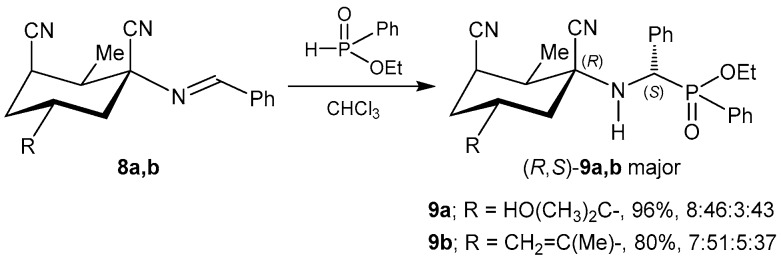
Nucleophilic addition of ethyl phenylphosphinate onto the chiral imines **8a**,**b**.

Rossi et al. [41] carried out the nucleophilic addition of the racemic ethyl phenylphosphinate onto the chiral imines **8a**,**b** in chloroform at 60–70 °C, to obtain the α-amino-*C*-phosphinates **9a**,**b** in 96% and 80% yield, respectively, and good diastereoselectivities (Scheme 6).

**Scheme 7:**
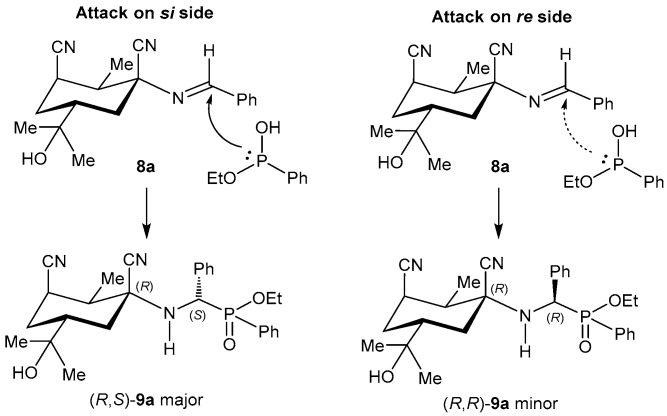
Preferential addition of ethylphenylphosphinate onto the *si* face of the imine **8a**.

The (*S*)-configuration at C_α_ of the major diastereoisomers **9a** resulted from the preferred addition of ethyl phenylphosphinate onto the *si* face of the imine **8a**, which adopts the most stable conformation according to the 1,3-allylic strain model [37], as shown in the Scheme 7 [41].

**Scheme 8:**
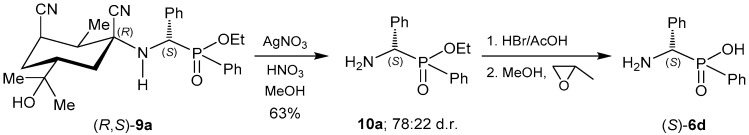
Synthesis of the (*S*)-α-aminobenzyl phenylphosphinic acid **6d**.

The reaction of (*R*,*S*)-**9a** with aqueous AgNO_3_/HNO_3_ in methanol at 50 °C, produced the α-amino-*C*-phosphinate **10a** in 63% yield and with 78:22 diastereoisomeric ratio, which by treatment with HBr/AcOH, afforded the enantiomerically pure (*S*)-α-aminobenzyl phenylphosphinic acid **6d** (Scheme 8) [41].

**Scheme 9:**
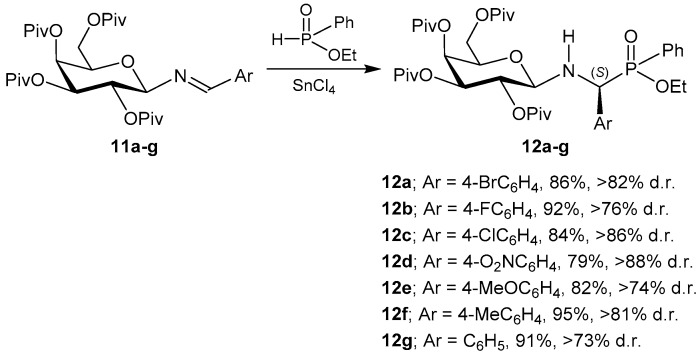
Nucleophilic addition of ethyl phenylphosphinate to *N*-galactosylaldimines **11a**–**g**.

Carbohydrate derivatives are efficient auxiliaries in many stereoselective chiral syntheses [42,43,44]. For example, Chen et al. [45] achieved the nucleophilic addition of ethyl phenylphosphinate to *N*-galactosylaldimines **11a**–**g** in the presence of catalytic amounts of SnCl_4_ in THF at room temperature to obtain the *N*-galactosyl α-aminoalkyl-*C*-phosphinates **12a**–**g** in good yield and moderate diastereoselectivity (Scheme 9). Diastereoisomerically pure compounds **12a**–**c**,**e**,**g** were obtained by recrystallization.

**Scheme 10:**
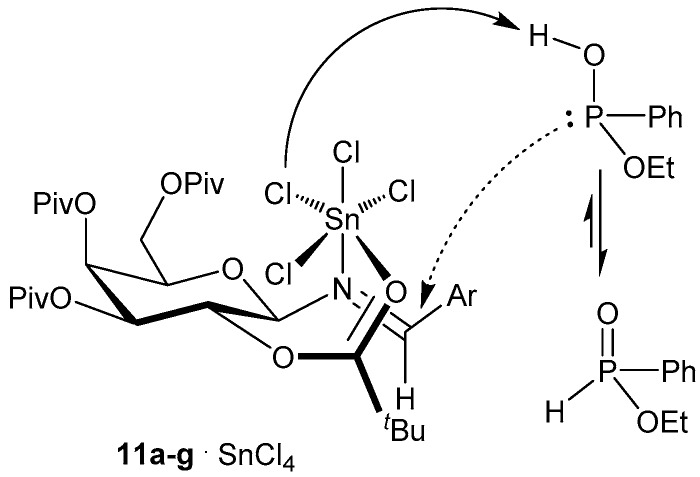
Plausible reaction mechanism for the addition of ethyl phenylphosphinate to **11a**–**g**.

The (*S*)-configuration at the α-carbon of the major diastereoisomers **12a**–**g** can be explained by the attack of ethyl phenylphosphinate from *si* face of (*E*)-imines **11a**–**g**. The imine-SnCl_4_ complex and simultaneous chelation with the auxiliary’s pivaloyl group inhibits free rotation along the *N*-anomeric carbon bond. According to this rationalization, the attack of the phosphinate in its P^III^ phosphonous tautomeric form proceeds from the back face of the imine **11a**–**g**, as shown in Scheme 10 [45].

**Scheme 11:**
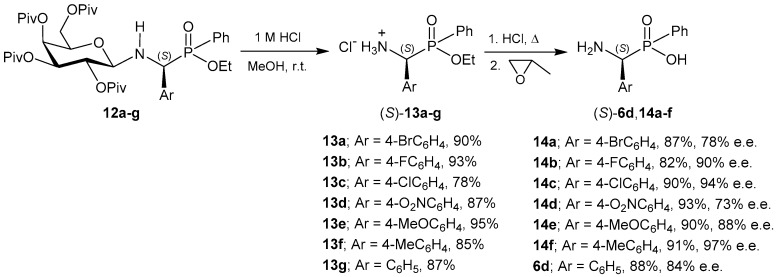
Synthesis of the α-amino-*C*-phosphinic acids **6d** and **14a**–**f**.

With the aim to assign the configuration, the reaction of *N*-galactosyl α-aminoalkyl-*C*-phosphinates **12a**–**g** with 1 M HCl/MeOH at room temperature produced the α-amino-*C*-phosphinate hydrochlorides **13a**–**g** in 78%–95% yield, which by hydrolysis with 1.5 M HCl under reflux followed by treatment with propylene oxide in ethanol at reflux, afforded the α-amino-*C*-phosphinic acids **6d** and **14a**–**f** in 82%–93% yield and with 73%–97% enantiomeric excess (Scheme 11) [45].

**Scheme 12:**
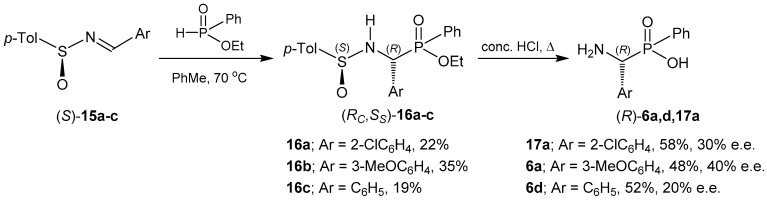
Synthesis of α-amino-*C*-phosphinic acids from chiral *N*-sulfinylaldimines (*S*)-**15a**–**c**.

Readily available enantiopure sulfinylimines also constitute valuable molecules in asymmetric synthesis [46]. Indeed, Petneházy et al. [47] reported the synthesis of optically enriched α-amino-*C*-phosphinic acids using ethyl phenylphosphinate and chiral *N*-sulfinylaldimines. In this context, the nucleophilic addition of ethyl phenylphosphinate to the chiral imines (*S*)-**15a**–**c** in toluene at 70 °C, furnished the α-amino-*C*-phosphinates (*R*_C_,*S*_S_)-**16a**–**c** in 19%–35% yield, which by simultaneous removal of the *N*-sulfinyl auxiliary and hydrolysis of the ethyl phosphinate with concentrated HCl at reflux, gave the α-amino-*C*-phosphinic acids (*R*)-**6a**,**d** and (*R*)-**17a** in 48%–58% yield and 20%–40% enantiomeric excesses (Scheme 12).

#### 2.1.2. Chiral Catalyst

**Scheme 13:**
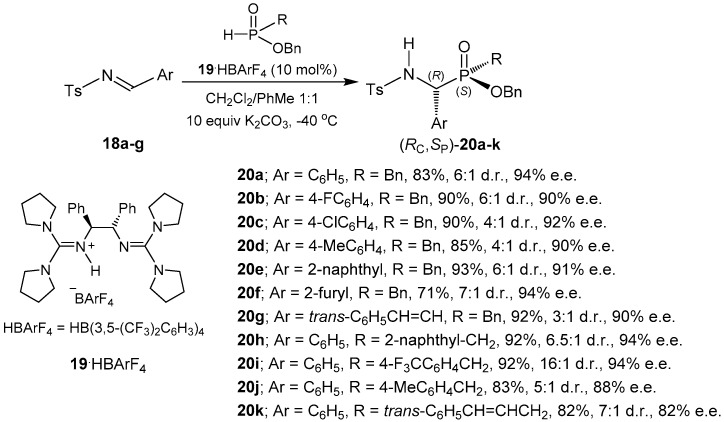
Hydrophosphinylation of aldimines **18a**–**g** under organocatalytic conditions.

The stereoselective hydrophosphinylation of aldimines under organocatalytic conditions is emerging as a suitable method for the synthesis of optically active α-amino-*C*-phosphinates, in this regard the guanidines and guanidinium salts have shown to be highly efficient catalysts for enantioselective reactions [48]. For example, following the principle of activation of imines through hydrogen bonding, Tan et al. [49] carried out the hydrophosphinylation reaction of the *N*-tosyl imines **18a**–**g** with arylphosphinates and alkylphosphinates in the presence of catalytic amounts of the guanidinium salt **19**·HBArF_4_ in a CH_2_Cl_2_:toluene mixture and K_2_CO_3_ as a base at −40 °C, obtaining the α-amino-*C*-phosphinates **20a**–**k** in 71%–93% yield and 3:1–16:1 diastereoisomeric ratio, with preference for the diastereoisomers (*R*_C_,*S*_P_)-**20a**–**k** with 82%–94% enantiomeric excess (Scheme 13).

### 2.2. Stereoselective C-P Bond Formation (One-Pot Three-Component Reaction)

#### 2.2.1. Chiral Amino Compounds

Possibly the simplest experimental proposal to prepare optically active α-amino-*C*-phosphinates is the “one-pot” three-component reaction, where the reactants are placed all together with or without solvent and catalyst, which is identical to the Kabachnik-Fields reaction widely studied in the synthesis of α-aminophosphonates [50,51,52].

**Scheme 14:**
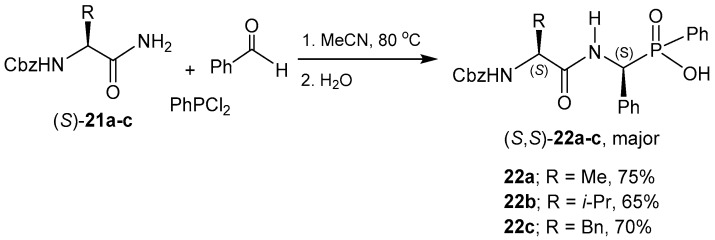
Preparation of phosphinopeptides (*S*,*S*)-**22a**–**c**.

Meng and Xu [19] reported a direct synthetic route for the preparation of phosphinopeptides containing *C*-terminal α-aminoalkyl-*C*-phosphinic acids, valuable peptide mimics. Thus, the “one-pot” three-component reaction of *N*-Cbz-aminoalkanamides (*S*)-**21a**–**c** obtained from *N*-protected amino acids, with benzaldehyde and phenyldichlorophosphine in dry acetonitrile at 80 °C followed by hydrolysis, produced the phosphinopeptides **22a**–**c** in 65%–75% yield and low degree of asymmetric induction, obtaining the (*S*,*S*)-**22a**–**c** diastereoisomers as major products (Scheme 14).

**Scheme 15:**
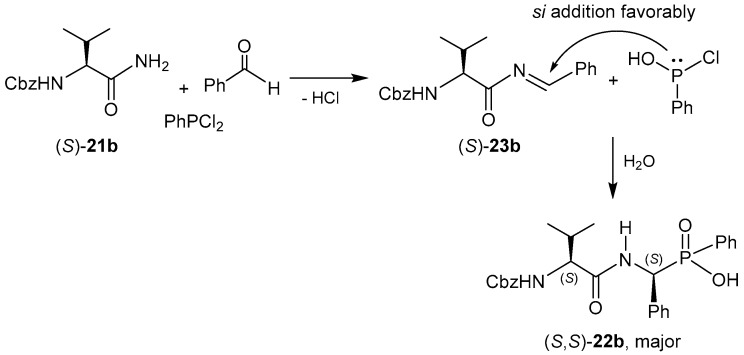
Mechanism for the formation of diastereoisomeric phosphinopeptide (*S*,*S*)-**22b**.

According to the authors, the reaction of the *N*-Cbz-aminoalkanamide, benzaldehyde and phenyldichlorophosphine produce the *N*-acylimine **23b** and phenylchlorophosphonous acid, which attacks to the imine favorably from the *si* face because the amino acid substituent blocks the opposite *re* face. Finally, a proton transfer affords the intermediate aminoalkylphosphinic chloride, which undergoes hydrolysis gave the (*S*,*S*)-phosphinopeptide **22b** as major product (Scheme 15) [19].

**Scheme 16:**
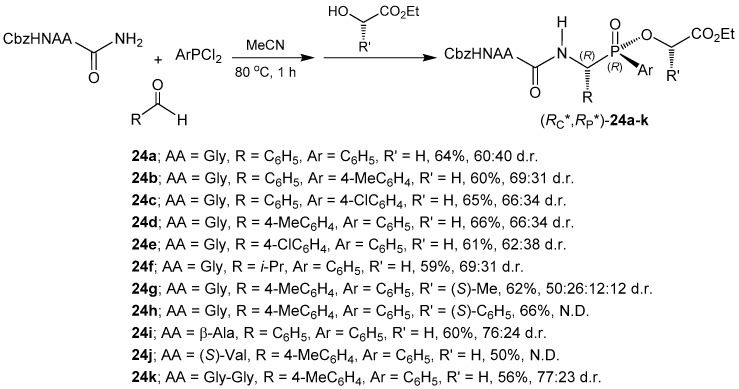
Synthesis of phosphinodepsipeptides (*R*_C_*,*R*_P_*)-**24a**–**k**.

Similarly, a series of phosphinodepsipeptides were synthesized via a pseudo-four-component condensation reaction, in a convergent and atom economic synthetic strategy [53]. In this context, the “one-pot” reaction of *N*-Cbz-amino amides derived from amino acids with different aldehydes and aryldichlorophosphines in dry acetonitrile at 80 °C followed by alcoholysis with α-hydroxy esters or ethanol, produced the phosphinodepsipeptides **24a**–**k** in 50%–66% yield and low diastereo selectivities, where the (*R*_C_*,*R*_P_*)-products were assumed as major diastereoisomers (Scheme 16).

**Scheme 17:**
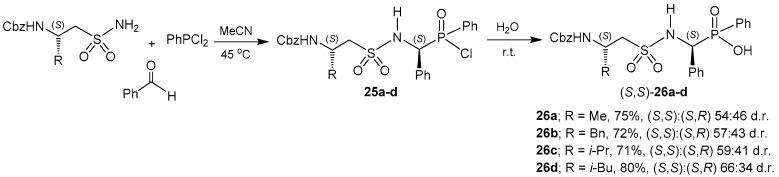
Synthesis of the sulfonophosphinodipeptides **26a**–**d**.

In a similar way, Meng et al. [54] reported the preparation of hybrid sulfonophosphinopeptides incorporating α-aminoalkyl-*C*-phosphinic acids. The compounds of interest were synthesized by reaction of the *N*-Cbz protected aminoalkanesulfonamides, benzaldehyde and phenyldichloro phosphine in dry acetonitrile at 45 °C and subsequent hydrolysis at room temperature, obtaining the sulfonophosphinodipeptides **26a**–**d** in good yield, but low diastereoselectivity. The diastereo isomers (*S*,*S*)-**26a**–**d** were assumed as the major products (Scheme 17).

### 2.3. Stereoselective C-H Bond Formation

#### 2.3.1. Reduction of C=C Bond in α,β-Dehydroaminophosphinates

**Scheme 18:**
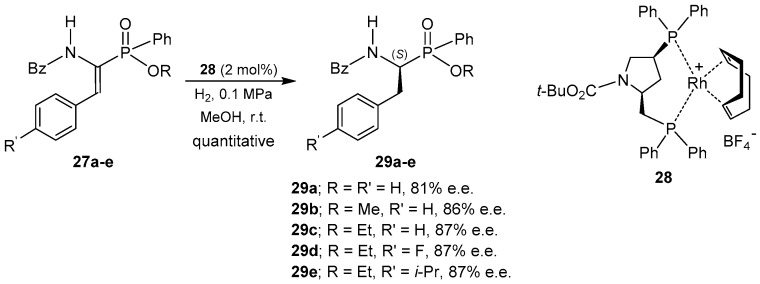
Catalytic asymmetric hydrogenation of the α,β-dehydroaminophosphinates **27a**–**e**.

Catalytic asymmetric hydrogenation of α,β-dehydroaminophosphinates is another nice method for the synthesis of optically pure α-amino-*C*-phosphinic acids and its derivatives. For example, Drauz et al. [55] described for the first time the catalytic asymmetric hydrogenations of the α,β-dehydroaminophosphinates **27a**–**e** [56] using the rhodium complex **28** in methanol at room temperature and 0.1 MPa H_2_-pressure, obtaining the α-amino-*C*-phosphinates **29a**–**e** in quantitative yield and 81%–87% enantiomeric excesses (Scheme 18).

**Scheme 19:**
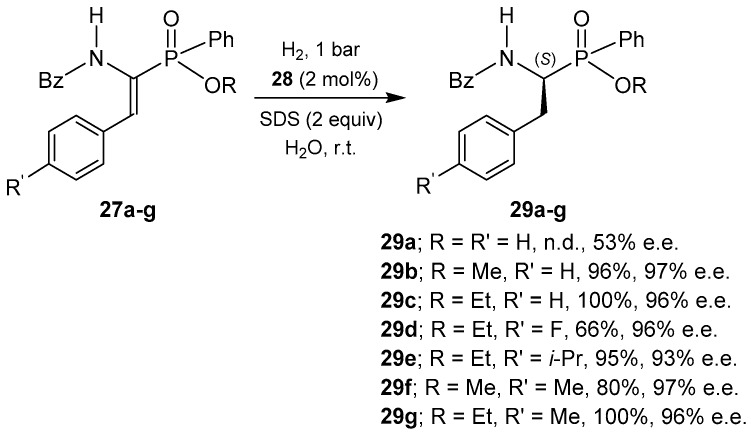
Optimized conditions for the catalytic hydrogenation of **27a**–**g**.

Additionally, Oehme et al. [57] conducted a comprehensive study on the hydrogenation of α,β-dehydroaminophosphinates evaluating different chiral rhodium complexes, substrate-catalyst ratio, temperatures, hydrogen pressures, modified substrates and both organic and aqueous solvents. Thus, under optimized conditions, they carried out the catalytic hydrogenation of the α,β-dehydroaminophosphinates **27a**–**g** [56] in the presence of catalytic amounts of [Rh(cod)(*S*,*S*)-bppm]BF_4_ **28** in aqueous micellar media at room temperature and 1 bar H_2_-pressure, obtaining the α-amino-*C*-phosphinates **29a**–**g** in 66%–100% yield and with 53%–97% enantiomeric excess (Scheme 19).

**Scheme 20:**
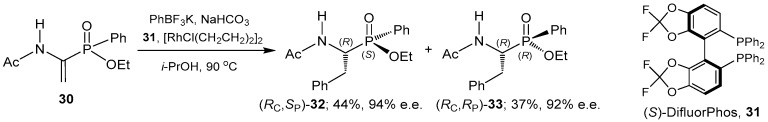
Asymmetric rhodium-catalyzed addition of phenyltrifluoroborate to **30**.

Furthermore, Darses et al. [58] reported an alternative approach for the synthesis of α-amino-*C*-phosphinates through the asymmetric rhodium-catalyzed addition of organoboron derivatives to α,β-dehydroaminophosphinates. Indeed, the addition of potassium phenyltrifluoroborate to the α,β-dehydroaminophosphinate **30** [59] in the presence of NaHCO_3_, [RhCl(CH_2_CH_2_)_2_]_2_ as catalyst precursor and (*S*)-DifluorPhos **31** as chiral ligand in isopropyl alcohol at 90 °C, afforded the diastereoisomeric α-amino-*C*-phosphinates **32** and **33** in moderate yield and with 94% and 92% enantiomeric excesses, respectively (Scheme 20).

### 2.4. Resolution Methodologies

**Scheme 21:**
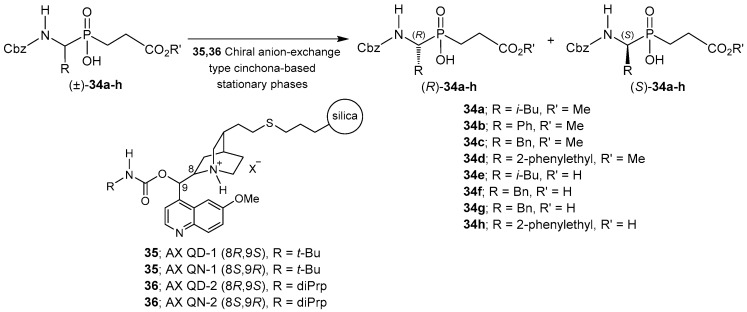
Chiral HPLC separation of *N*-Cbz phosphinic pseudopeptides **34a**–**h**.

The resolution strategies of racemic compounds have also demonstrated its usefulness for the preparation of optically enriched α-amino-*C*-phosphinic acids. For example, Lämmerhofer et al. [60] carried out the chiral HPLC separation of *N*-Cbz phosphinic pseudopeptide esters **34a**–**d** as well as their free C-terminal carboxylic derivatives **34e**–**h** using a set of cinchona alkaloid-derived chiral anion-exchangers **35**,**36** (Scheme 21). Semi-preparative scale chromatography supplied single enantiomers in 100 mg quantities allowing the biological activity assays.

**Scheme 22:**
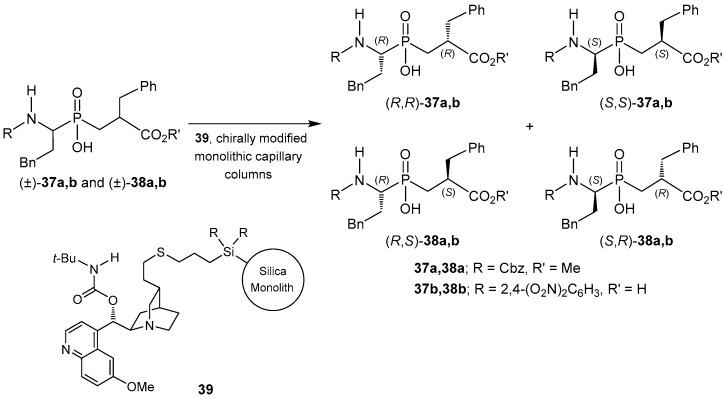
Capillary electrochromatography (CEC) method for the separation of (±)-**37a**,**b** and (±)-**38a**,**b**.

In a related work, Lämmerhofer et al. [61] developed a capillary electrochromatography (CEC) method for the separation of the stereoisomers of *N*-Cbz phosphinic pseudodipeptide methyl ester **37a** and **38a** and its *N*-DNP derivative with free *C*-terminal carboxylic group **37b** and **38b**, using a monolithic silica capillary column modified with a cinchona alkaloid-derived anion-exchange-type chiral selector **39** (Scheme 22). This method proved superiority compared to the HPLC separation, which was attributed to significantly enhanced plate numbers.

**Scheme 23:**
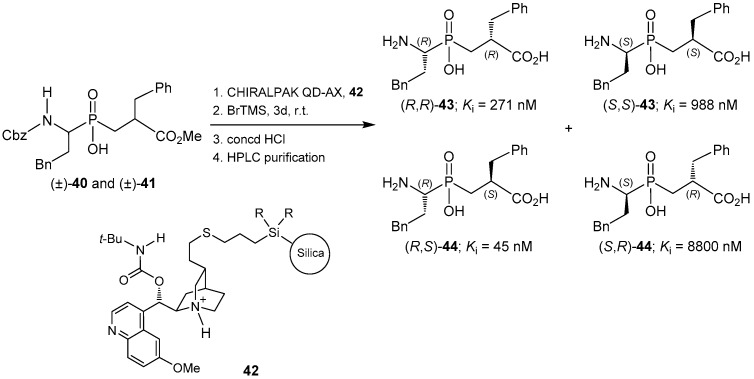
Chiral HPLC separation of phosphinic dipeptide derivatives **40** and **41** and activities of unprotected derivatives **43** and **44** as inhibitors of leucine aminopeptidase (LAP).

Taking into account that phosphinic dipeptides, although tested as stereoisomeric mixtures, are considered amongst the most potent inhibitors of bizinc cytosolic leucine aminopeptidase (LAP) [62], Mucha, et al. [63] carried out the chiral HPLC separation of all stereoisomers of phosphinic dipeptide homophenylalanyl-phenylalanine derivative **40** and **41** on a quinidine carbamate modified silica stationary phase **42** (Scheme 23). Significant differences in inhibition of the unprotected derivatives **43** and **44** show the importance of the molecular stereochemistry in spatial interaction with the enzyme active site.

**Scheme 24:**
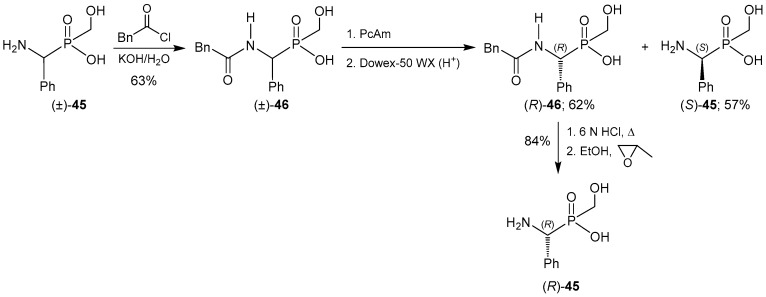
Synthesis of optically pure α-aminobenzyl hydroxymethyl phosphinic acid (*R*)- and (*S*)-**45**.

In another example, Ragulun and Rozhko [64] reported the preparation of the optically pure α-aminobenzyl hydroxymethyl phosphinic acid (*R*)- and (*S*)-**45**, where the key step was a biocatalytic resolution. Thus, the racemic α-aminobenzyl hydroxymethyl phosphinic acid (±)-**45**, obtained from the hydroxymethyl phosphinic acid, was acylated with phenylacetyl chloride and KOH in water to obtain the *N*-phenylacetyl derivative **46** in 63% yield, which by hydrolysis using penicillin amidase (PcAm) as the biocatalyst, afforded the enantiomerically pure (*R*)-**46** in 62% yield and the deacylated product (*S*)-**45** in 57% yield. Finally, hydrolysis of compounds (*R*)-**46** with 6 N HCl at reflux followed by treatment with propylene oxide, provided the α-amino-*C*-phosphinic acid (*R*)-**45** in 84% yield (Scheme 24).

### 2.5. Conversion from Chiral α-Aminophosphonates

**Scheme 25:**
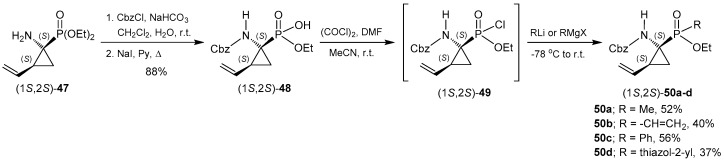
Transformation of chiral phosphonate (1*S*,2*S*)-**47** into *C*-phosphinates (1*S*,2*S*)-**50a**–**d**.

Undoubtedly, a useful but not as explored strategy for the synthesis of optically enriched α-amino-*C*-phosphinic acids is their conversion from the corresponding enantiomerically pure α-aminophosphonic counterparts [8]. Considering this possibility, Pyun et al. [65,66] carried out the transformation of chiral phosphonate (1*S*,2*S*)-**47** into *C*-phosphinates (1*S*,2*S*)-**50a**–**l**. Thus, the reaction of the optically pure diethyl (1*S*,2*S*)-1-amino-2-vinylcyclopropanephosphonate **47** [67] with benzyl chloroformate and NaHCO_3_ followed by treatment with NaI in pyridine at reflux, gave the phosphonate monoester (1*S*,2*S*)-**48** in 88% yield. The reaction of (1*S*,2*S*)-**48** with oxalyl chloride and DMF in MeCN, afforded the phosphonomonochloridate (1*S*,2*S*)-**49**, that without additional purification was reacted with different organolithium or Grignard reagents, to obtain the α-amino-*C*-phosphinates (1*S*,2*S*)-**50a**–**d** in 37%–56% yield, which have been used in the design and synthesis of new enzyme inhibitors (Scheme 25).

**Scheme 26:**
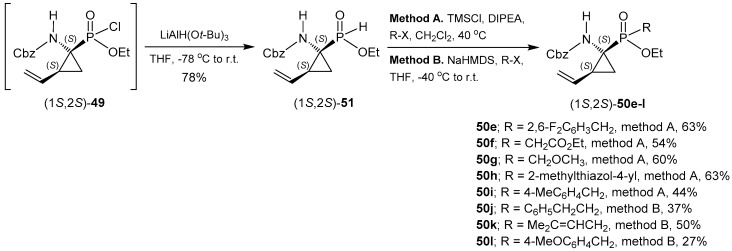
Transformation of chiral phosphonate (1*S*,2*S*)-**47** into *C*-phosphinates (1*S*,2*S*)-**50e**–**l**.

On the other hand, the reduction of the phosphonomonochloridate (1*S*,2*S*)-**49** with LiAlH(O*t*-Bu)_3_ in THF at −78 °C, produced the *H*-phosphinate (1*S*,2*S*)-**51** in 78% yield, which by alkylation either by activation with chlorotrimethylsilane (TMSCl) and diisopropyl ethylamine (DIPEA) followed by addition of an alkyl halide (method A), or using NaHMDS and subsequent addition of an alkyl halide (method B), furnished the *P*-alkylated α-amino-*C*-phosphinates (1*S*,2*S*)-**50e**–**l** in 27%–63% yield (Scheme 26) [65,66].

**Scheme 27:**
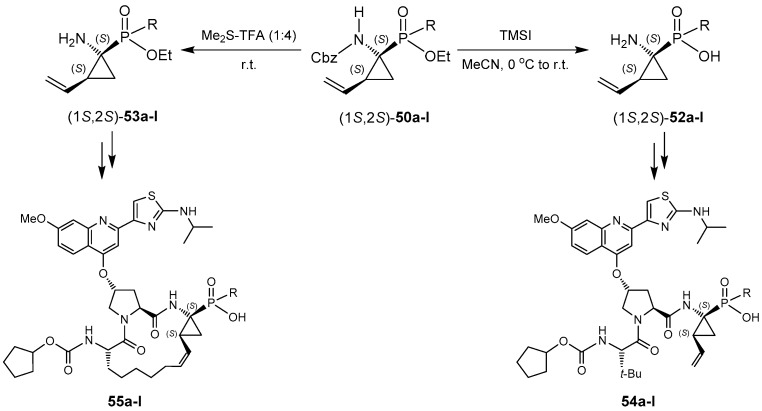
Synthesis of the acyclic **54a**–**l** and cyclic **55a**–**l** *C*-phosphinate analogs of BI-2061.

Additionally, the reaction of (1*S*,2*S*)-**50a**–**l** with iodotrimethylsilane in MeCN produced the simultaneous deprotection of both the Cbz group and the ethyl ester, to obtain the α-amino-*C*-phosphinic acids (1*S*,2*S*)-**52a**–**l**. On the other hand, cleavage of *N*-Cbz bond in (1*S*,2*S*)-**50a**–**l** with TFA-SMe_2_ [68] gave the α-amino-*C*-phosphinates (1*S*,2*S*)-**53a**–**l**. Both, the α-amino-*C*-phosphinic acids (1*S*,2*S*)-**52a**–**l** and *C*-phosphinates (1*S*,2*S*)-**53a**–**l** were used in the synthesis of the acyclic **54a**–**l** and cyclic **55a**–**l** *C*-phosphinate analogs of BI-2061 [66], respectively, and their inhibition of the HCV NS3 protease was tested (Scheme 27).

**Figure 3:**
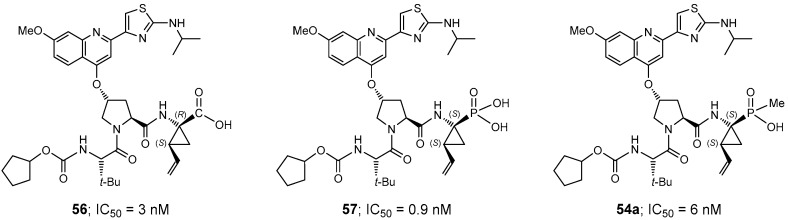
Carboxylic **56**, phosphonic **57** and *C*-phosphinic **54a** analogs of BI-2061 and activities as inhibitors of the HCV NS3 protease.

The acyclic *C*-phosphinate analog **54a** showed an IC_50_ of 6 nM, whereas the carboxylic **56** and phosphonic **57** derivatives were found to have an IC_50_ of 3 and 0.9 nM, respectively. The lower inhibition of the *C*-phosphinic compared to its phosphonic analog was attributed to the loss of *H*-bonding interaction (Figure 3) [66].

**Scheme 28:**
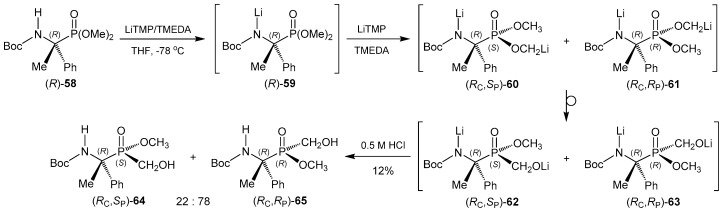
LiTMP induced phosphonate-phosphinate rearrangement of the α-aminophosphonate (*R*)-**58**.

Recently, Hammerschmidt et al. [69] reported the *s*-BuLi or LiTMP induced phosphonate-phosphinate rearrangement of *N*-Boc α-aminophosphonates through deprotonation- metallation at the methoxy group. In this context, the treatment of enantiomerically pure *N*-Boc α-aminophosphonate **58** with 2.5 equiv of LiTMP in dry THF at −78 °C followed by acidic workup, provided the *C*-phosphinates (*R*,*S*_P_)-**64** and (*R*,*R*_P_)-**65** in 12% yield and with 22:78 diastereoisomeric ratio, via the metallated phosphonates (*R*_C_,*S*_P_)-**60** and (*R*_C_,*R*_P_)-**61** and the ensuing rearrangement to *C*-phosphinates (*R*_C_,*S*_P_)-**62** and (*R*_C_,*R*_P_)-**63**, respectively (Scheme 28).

### 2.6. P-C Bond Formation from Chiral α-Amino-H-phosphinic Derivatives

**Scheme 29:**
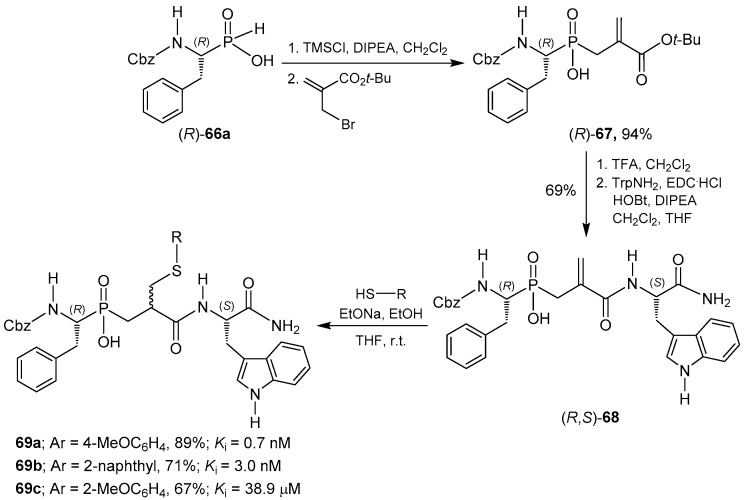
Synthesis of phosphinic pseudotripeptides **69a**–**c** and their activities toward MMP-8 (human neutrophile collagenase, HNC).

There is a continuously growing interest in phosphorus-carbon bond formation due to the great applications of organophosphorus compounds in synthetic organic and bioorganic chemistry [5,70]. In this context, the chiral α-amino-*H*-phosphinates have also proved efficiency as *P*-nucleophiles in Michael additions. In this regard, Yiotakis et al. [27,71] reported the synthesis of dehydroalaninyl phosphinic dipeptide analogues, which were tested as zinc-metalloproteases MMP-8 inhibitors. For this purpose, the optically pure α-amino-*H*-phosphinic acid (*R*)-**66a** [72] was reacted with TMSCl/DIPEA in CH_2_Cl_2_ followed by addition of *tert*-butyl 2-(bromomethyl)acrylate, obtaining the dehydroalanine derivative (*R*)-**67** in 94% yield, presumably via an allylic rearrangement. Cleavage of the *O*-*tert*-butyl bond in (*R*)-**67** with trifluoroacetic acid (TFA) and coupling with l-tryptophanyl-amide, gave the pseudotripeptide (*R*,*S*)-**68** in 69% yield. Finally, the Michael-type addition of benzyl thiols to (*R*,*S*)-**68**, produced the cysteine analogues **69a**–**c** in good yield (Scheme 29).

**Scheme 30:**
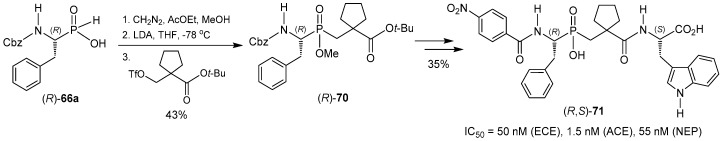
Synthesis of the phosphinic pseudotripeptide (*R*,*S*)-**71** and its activities as inhibitor of endothelin converting enzyme (ECE), angiotensin converting enzyme (ACE) and neutral endopeptidase (NEP).

On the other hand, McKittrick et al. [73] reported the synthesis of the phosphinic pseudotripeptide (*R*,*S*)-**71**, which not only inhibits the metalloprotease endothelin converting enzyme (ECE), but also the angiotensin converting enzyme (ACE) and neutral endopeptidase (NEP) with IC_50_ values <100 nM. Thus, the hydroxyphosphinyl group in enantiopure Cbz-protected phenylalanine phosphinic analogue (*R*)-**66a** [72] was reacted with diazomethane, followed by treatment with LDA in THF at −78 °C and subsequent addition of the triflate, to afford the *P*-alkylated α-amino-*C*-phosphinate (*R*)-**70** in 43% yield, which was further transformed into pseudotripeptide (*R*,*S*)-**71** (Scheme 30).

**Scheme 31:**
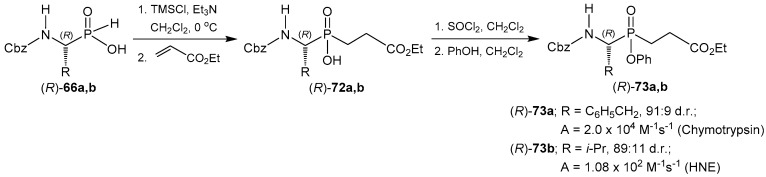
Synthesis of phenyl phosphinate derivatives **73a**,**b** and second-order rate constants [A = k/K_i_] of inhibition of chymotrypsin and human neutrophile elastase (HNE).

Hamilton et al. [74] reported the synthesis of phenyl phosphinate derivatives **73a**,**b** from Cbz-protected phenylalanine and valine phosphinic analogues [72] and their inhibitory properties against human neutrophile elastase (HNE) (valine analogue) and chymotrypsin (phenylalanine analogue) were evaluated, determining specific inhibitory activity for analogues having (*R*) configuration at α-carbon. Thus, the reaction of the α-amino-*H*-phosphinic acids (*R*)-**66a**,**b** with TMSCl and Et_3_N in CH_2_Cl_2_ at 0 °C followed by the addition of ethyl acrylate, produced the Michael adducts (*R*)-**72a**,**b**, which by reaction with thionyl chloride (SOCl_2_) in CH_2_Cl_2_ followed by addition of phenol, provided the phenyl *C*-phosphinates (*R*)-**73a**,**b** with 91:9 and 89:11 diastereoisomeric ratio, respectively (Scheme 31).

**Scheme 32:**
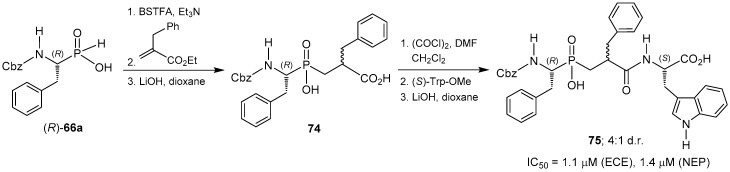
Synthesis of the *C*-phosphinic acid **75** and its activities as inhibitor of endothelin converting enzyme (ECE) and neutral endopeptidase (NEP).

Additionally, Lloyd et al. [75] synthesized the *C*-phosphinic acid **75** and tested its effect on ECE inhibition. In this context, the reaction of α-amino-*H*-phosphinic acid (*R*)-**66a** obtained by resolution [72], with *N*,*O*-bis(trimethylsilyl)trifluoroacetamide (BSTFA) followed by the Michael addition to ethyl 2-benzylacrylate and subsequent basic hydrolysis of the ethoxycarbonyl group, produced the *C*-phosphinic acid **74**. Finally, the reaction of **74** with oxalyl chloride, followed by coupling with (*S*)-Trp-OMe and subsequent saponification with LiOH, afforded the phosphinic pseudopeptide **75** as a 4:1 diastereoisomeric mixture (Scheme 32).

**Scheme 33:**
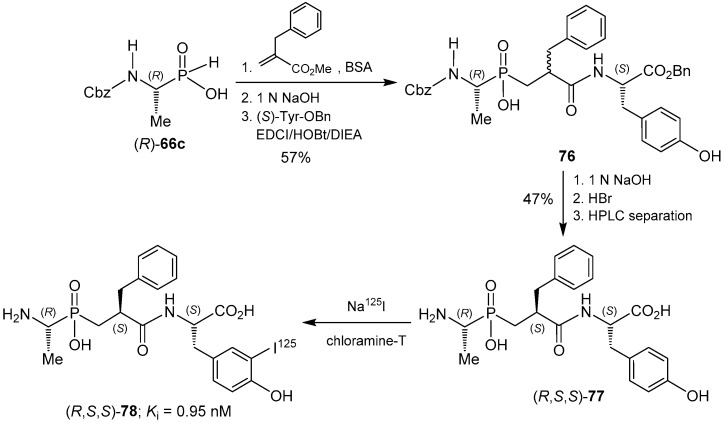
Synthesis of the iodinated tripeptide (*R*,*S*,*S*)-**78** and its activity as inhibitor of aminopeptidase N.

Roques et al. [76] reported the synthesis of the iodinated tripeptide (*R*,*S*,*S*)-**78** analogue of a highly efficient aminopeptidase N inhibitor, as a tool for complete characterization of the biochemical and pharmacological properties of this enzyme. The Michael addition of *N*-Cbz alanine *H*-phosphinic analogue (*R*)-**66c** obtained by resolution [72], to the methyl benzylacrylate in the presence of *N*,*O*-bistrimethylsilylacetamide (BSA), followed by alkaline hydrolysis and subsequent coupling with the l-tyrosine benzyl ester, produced the tripeptide **76** in 57% yield as a diastereoisomeric mixture, which by saponification of benzyl ester and Cbz removal under acidic conditions, gave the (*R*,*S*,*S*)-**77** diastereoisomer in 47% yield after HPLC separation. Finally, the radioiodination of (*R*,*S*,*S*)-**77** was performed by using Na^125^I in sodium hydroxide solution and chloramine-T, providing the radiolabeled compound (*R*,*S*,*S*)-**78** (Scheme 33).

**Scheme 34:**
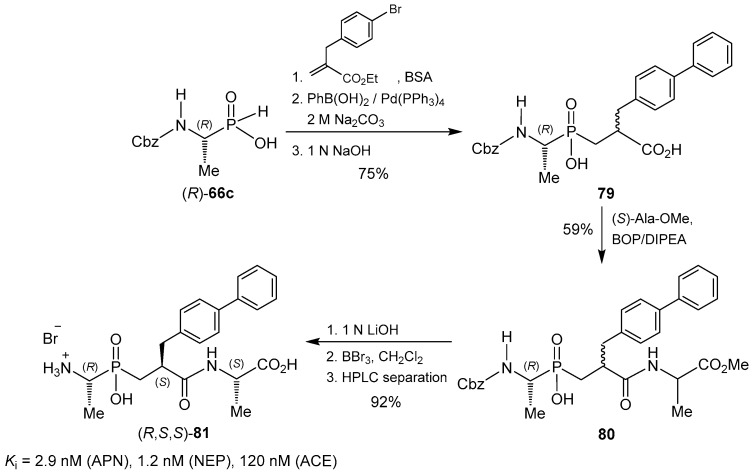
Synthesis of the phosphinic pseudopeptide (*R*,*S*,*S*)-**81** and its activities as inhibitor of aminopeptidase N (APN), neutral endopeptidase (NEP) and angiotensin converting enzyme (ACE).

In a similar way, Roques et al. [77] reported the synthesis of the phosphinic pseudopeptide (*R*,*S*,*S*)-**81**, which proved to be a potent dual inhibitor of the zinc metallopeptidases neprilysin and aminopeptidase N. For this purpose, the Michael addition of *N*-Cbz alanine *H*-phosphinic acid (*R*)-**66c** obtained by resolution [72], to ethyl *p*-bromobenzylacrylate followed by a Suzuki coupling using phenylboronic acid and subsequent alkaline hydrolysis of the ethyl ester, gave the *C*-phosphinic acid derivative **79** in 75% yield, which by the coupling with (*S*)-alanine methyl ester afforded the tripeptide **80** in 59% yield. Finally, the saponification of the methyl ester followed by the cleavage of the *N*-Cbz bond with BBr_3_ and the semipreparative HPLC purification, provided the enantiomerically pure phosphinic pseudopeptide (*R*,*S*,*S*)-**81** in 92% yield (Scheme 34).

**Scheme 35:**
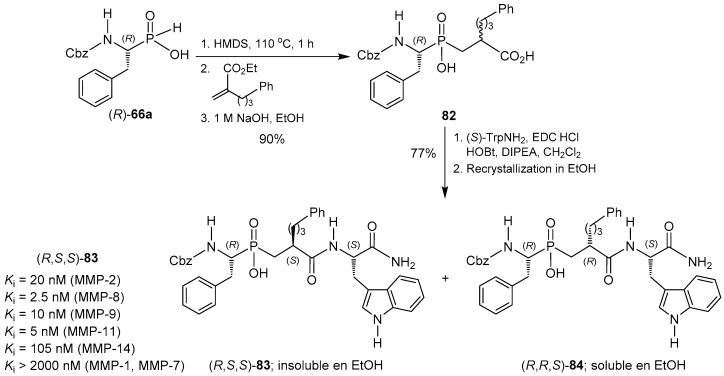
Synthesis of the phosphinic pseudotripeptides (*R*,*S*,*S*)-**83** and (*R*,*R*,*S*)-**84** and its activities of the most potent diastereoisomer of RXP03 [(*R*,*S*,*S*)-**83**] as inhibitor of matrix metalloproteinases.

In a related work, Samios et al. [78] developed an efficient synthetic methodology for the preparation of the phosphinic pseudotripeptide known as RXP03, which has been widely studied as MMPs inhibitor [79,80]. Initially, reaction of the *N*-Cbz *H*-phosphinic acid analogue of phenylalanine (*R*)-**66a** [72] with bis(trimethylsilyl)amine (HMDS) followed by the addition of ethyl phenylpropyl-acrylate and subsequent hydrolysis of the ethyl ester group, gave *C*-phosphinate **82** in 90% yield, which by coupling with (*S*)-TrpNH_2_, afforded the phosphinic pseudotripeptides (*R*,*S*,*S*)-**83** and (*R*,*R*,*S*)-**84** in 77% yield, which were separated in 99% purity by preferential precipitation in EtOH (Scheme 35). This methodology allows the separation of the diastereoisomers based in their solubility differences, which was explained by conformational and solvation theoretical studies in terms of their intra- and intermolecular structure.

**Scheme 36:**
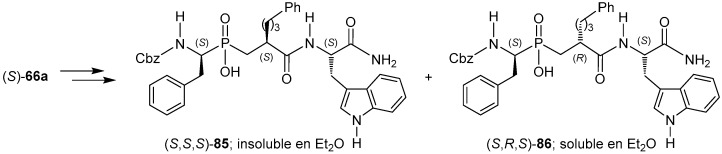
Synthesis of the phosphinic pseudotripeptides (*S*,*S*,*S*)-**85** and (*S*,*R*,*S*)-**86**.

Similarly, the phosphinic pseudotripeptides (*S*,*S*,*S*)-**85** and (*S*,*R*,*S*)-**86** were obtained from the enantiomeric pure *N*-Cbz *H*-phosphinic acid analogue of phenylalanine (*S*)-**66a** in 64% yield. As in the previous example, each diastereoisomer showed different solubility properties in ethyl ether allowing their complete separation (Scheme 36) [78].

**Scheme 37:**
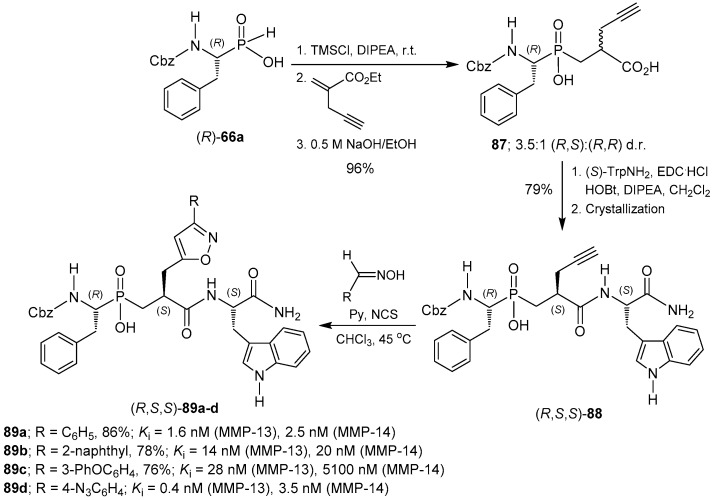
Preparation of a novel class of isoxazole-containing phosphinic peptides **89a**–**d** and its activities as inhibitors of matrix metalloproteinases MMP-13 and MMP-14.

Considering that phosphinic peptides are well recognized as peptide isosters and powerful inhibitors of many classes of enzymes, mainly zinc proteases, Yiotakis et al. [81,82] reported the synthesis of phosphinopeptidic building blocks incorporating a triple bond, which through 1,3-dipolar cycloaddition process, gives access to novel class of isoxazole-containing phosphinic peptides. Thus, the reaction of *N*-Cbz *H*-phosphinic acid analogue of phenylalanine (*R*)-**66a** [72] with TMSCl/DIPEA followed by the addition of ethyl α-propargylic acrylate and saponification of the ethyl ester, gave the *C*-phosphinates **87** in 96% yield and 3.5:1 diastereoisomeric ratio. Coupling of **87** with (*S*)-TrpNH_2_ afforded the tripeptide **88** as a diastereoisomeric mixture, from which the diastereoisomer (*R*,*S*,*S*)-**88** was isolated by means of simple crystallization. Finally, the 1,3-dipolar cycloaddition reaction of (*R*,*S*,*S*)-**88** with different nitrile oxides generated in situ from the chlorination of the corresponding oxime, furnished the isoxazoles (*R*,*S*,*S*)-**89a**–**d** in good yields (Scheme 37).

**Scheme 38:**
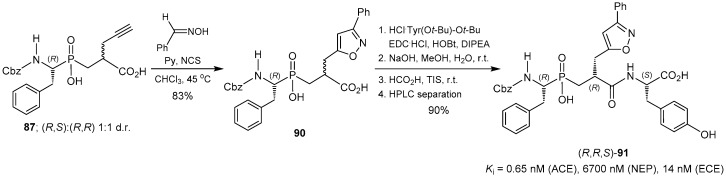
Synthesis of the phosphinic tripeptide (*R*,*R*,*S*)-**91** and its activities as inhibitor of angiotensin converting enzyme (ACE), neutral endopeptidase (NEP) and endothelin converting enzyme (ECE).

On the other hand, the 1,3-dipolar cycloaddition reaction of propargylic *C*-phosphinic acid **87** (1:1 diastereoisomeric mixture) [81] with benzonitrile oxide, gave the isoxazole-phosphinate **90** in 83% yield, which by coupling with di-*tert*-butyl protected (*S*)-tyrosine followed by the cleavage of the *tert*-butyl group and semipreparative RP-HPLC isolation, provided the pure diastereoisomeric phosphinic tripeptide (*R*,*R*,*S*)-**91** in 90% yield (Scheme 38) [83]. These compounds were administered intravenously and lowered mean arterial blood pressure in spontaneously hypertensive rats.

**Scheme 39:**
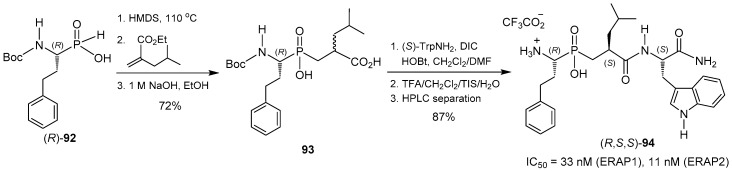
Synthesis of the phosphinic tripeptide (*R*,*S*,*S*)-**94** and its activities as inhibitor of intracellular aminopeptidases endoplasmic reticulum aminopeptidases 1 and 2 (ERAP 1 and ERAP 2).

In a similar way, Stratikos et al. [84] reported the synthesis of the phosphinic tripeptide (*R*,*S*,*S*)-**94**, which was tested in the inhibition of the endoplasmic reticulum aminopeptidases 1 and 2 (ERAP1 and ERAP2), involved in regulation of cytotoxic cellular responses. Thus, the reaction of *N*-Boc *H*-phosphinic acid analogue of homophenylalanine (*R*)-**92** [72] with HMDS followed by the addition of ethyl isobutylacrylate and saponification of the ethyl ester, gave the *C*-phosphinate **93** in 72% yield, which by coupling with (*S*)-TrpNH_2_, deprotection and HPLC separation, afforded the phosphinic tripeptide (*R*,*S*,*S*)-**94** in 87% (Scheme 39) [78].

**Scheme 40:**
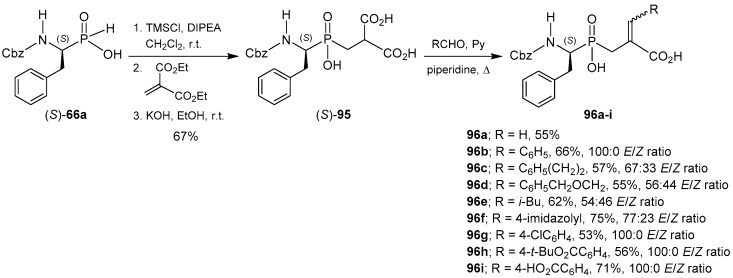
Synthesis of the dehydrophosphinic peptides **96a**–**i**.

In order to functionalize the phosphinic pseudopeptide framework, Yiotakis et al. [85] developed a simple and efficient method for P_1_′ diversification obtaining a variety of dehydrophosphinic peptides. According to this strategy, the treatment of *N*-Cbz phenylalanine *H*-phosphinic analogue (*S*)-**66a** [72] with TMSCl/DIPEA followed by addition of diethyl 2-methylenemalonate and subsequent alkaline hydrolysis, afforded the phosphinic pseudodipeptide (*S*)-**95** in 67% yield, which was subjected to a Knoevenagel-type condensation with several aldehydes, to give the dehydrophosphinic peptides **96a**–**i** in good yields (Scheme 40).

**Scheme 41:**
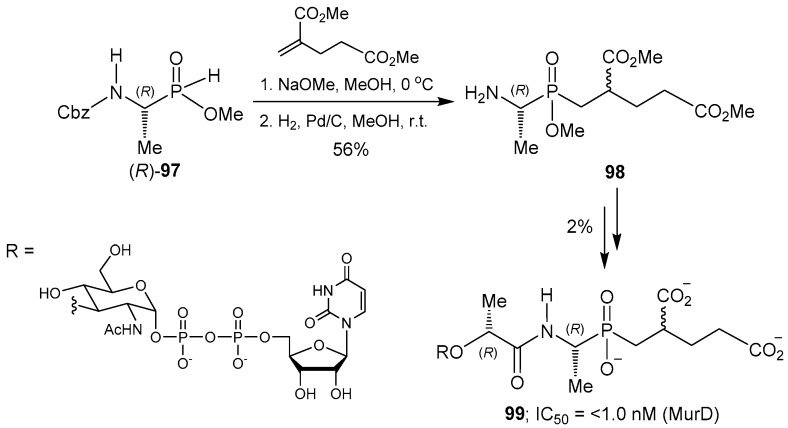
Synthesis of the phosphinic pseudopeptide **99** and its activity as inhibitor of the d-glutamic acid-adding enzyme (MurD) isolated from *E. coli*.

Gegnas et al. [86] described the synthesis of the phosphinic pseudopeptide **99**, which proved to be an inhibitor of the d-glutamic acid-adding enzyme (MurD) of bacterial peptidoglycan biosynthesis. The nucleophilic addition of compound (*R*)-**97** [72] to 2-methylene pentanedioate in the presence of sodium methoxide provided the dipeptide isostere as a mixture of four diastereoisomers, which under hydrogenolysis over Pd/C catalyst in methanol provided the derivative **98** with the free amino group in 56% yield. Compound **98** was further transformed into the target pseudopeptide **99** in 2% overall yield through a six steps sequence, whose biological activity lied below the sensitivity of the enzyme assay used (<1 nM) (Scheme 41).

**Scheme 42:**
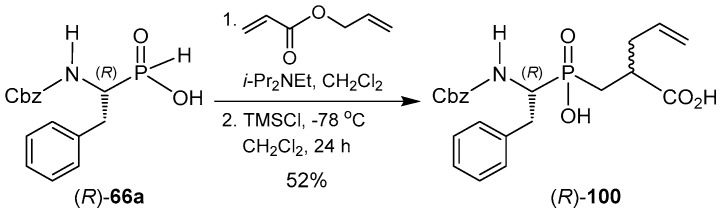
Synthesis of the α-amino-*C*-phosphinic acid (*R*)-**100**.

Additionally, Yiotakis et al. [87] developed an Ireland-Claisen rearrangement triggered by the phospha-Michael addition of silyl phosphonites to allyl acrylates, as a new strategy to access pharmacologically relevant *C*-phosphinic derivatives. In this context, the reaction of Cbz-protected phenylalanine *H*-phosphinic analogue (*R*)-**66a** [72] with the acrylate in the presence of DIPEA in CH_2_Cl_2_ followed by addition of TMSCl at −78 °C, provided the *P*-alkylated α-amino-*C*-phosphinic acid (*R*)-**100** in 52% yield with retention of configuration at phosphorus atom (Scheme 42).

**Scheme 43:**
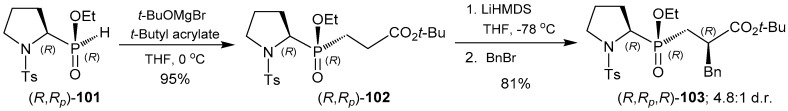
Synthesis of the Pro-Phe phosphinyl dipeptide (*R*,*R_P_*,*R*)-**103**.

On the other hand, the diastereoselective synthesis of Pro-Phe phosphinyl dipeptide isosteres was accomplished from optically active prolinephosphinate derivative (*R*,*R*_P_)-**101** [88], which was obtained in 13 steps from 1,1-diethoxyethyl(hydroxymethyl)phosphinate via a lipase-catalyzed acylation [89]. Thus, the Michael addition of the *H*-phosphinate (*R*,*R*_P_)-**101** to *t*-butyl acrylate in the presence of *t*-BuOMgBr in THF at 0 °C, provided the *C*-phosphinate (*R*,*R*_P_)-**102** in 95% yield, which by treatment with LiHMDS followed by the addition of benzyl bromide in THF at −78 °C, produced the Pro-Phe phosphinyl dipeptide (*R*,*R_P_*,*R*)-**103** in 81% yield and 4.8:1 diastereoisomeric ratio (Scheme 43).

## 3. Synthesis of Phosphacyclic α-Amino-C-phosphinates

Several strategies for the preparation of *P*-heterocycles have been described over the last years, and excellent reviews have been published [90,91,92,93]. In the past 20 years, significant effort has been devoted to synthetic and reactivity studies of this particular class of compounds, here we only report the stereoselective methods recently reported in the literature where the α-amino-*C*-phosphinate motif is incorporated.

### 3.1. 1,4,2-Oxazaphosphacycles

**Scheme 44:**
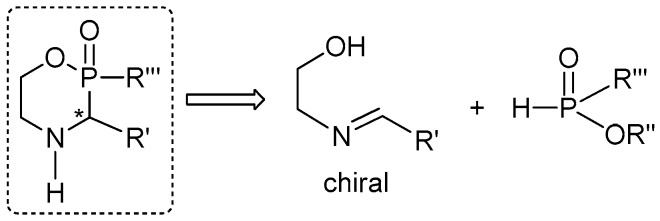
Retrosynthetic analysis of the 1,4,2-oxazaphosphacycle scaffold.

The strategies described for the stereoselective synthesis of 1,4,2-oxazaphosphacycles typically involve the diastereoselective nucleophilic addition-cyclization reaction from hypophosphorous acid (H_3_PO_2_) or methyl hypophosphite (H_2_PO_2_Me) and chiral imino alcohols (Scheme 44).

**Scheme 45:**
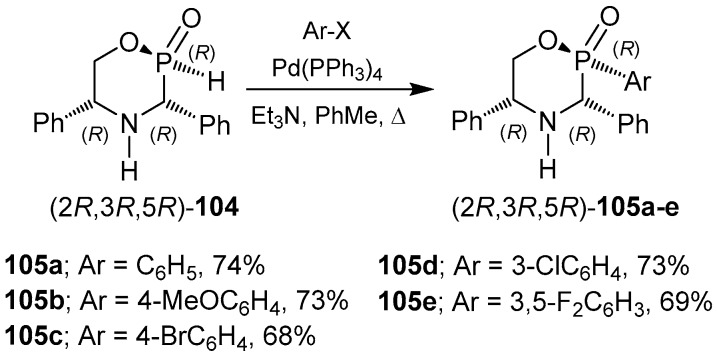
Palladium catalyzed coupling of (2*R*,3*R*,5*R*)-**104** with aryl halides.

With previously acquired knowledge in the preparation and reactivity of oxazaphosphinanes, Pirat et al. [94] addressed the enantioselective synthesis of phosphinic analogues of 2-aryl-morpholinols, which have shown strong activity on the noradrenergic systems [95] and therefore constitute new therapeutic means for depression treatment. Thus, the palladium catalyzed coupling of (2*R*,3*R*,5*R*)-**104** with aryl halides and catalytic amounts of tetrakis(triphenylphosphine) palladium and Et_3_N in PhMe at reflux, afforded the 2-aryl-1,4,2-oxazaphosphinanes (2*R*,3*R*,5*R*)-**105a**–**e** in 68%–74% yield as single diastereoisomers (Scheme 45). The retention of configuration at phosphorus atom was confirmed through X-ray analysis of final products.

**Scheme 46:**
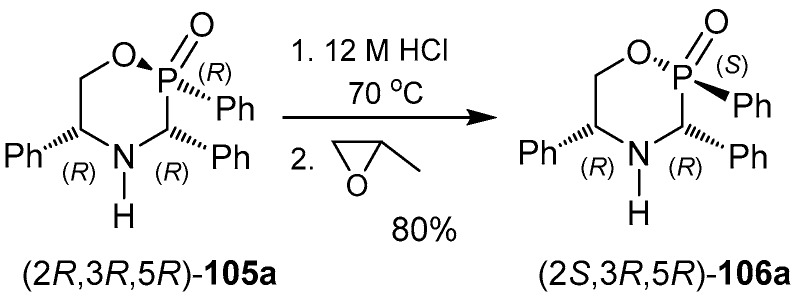
Inversion of configuration at the phosphorus of (2*R*,3*R*,5*R*)-**105a**.

Treatment of (2*R*,3*R*,5*R*)-**105a** with 12 M HCl at 70 °C and subsequent addition of propylene oxide, produced the (2*S*,3*R*,5*R*)-2,3,5-triphenyl-1,4,2-oxazaphosphinane (−)-**106a** in 80% yield through a selective inversion of configuration at phosphorus center (Scheme 46) [94].

**Scheme 47:**
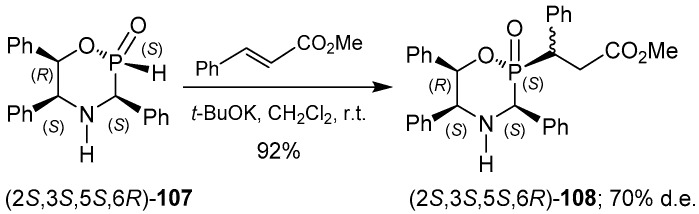
Michael addition of the oxazaphosphinane (2*S*,3*S*,5*S*,6*R*)-**107** to methyl cinnamate.

In a complementary work, Pirat et al. [96] evaluated the diastereoselectivity in the Michael addition of the enantiomerically pure oxazaphosphinane (2*S*,3*S*,5*S*,6*R*)-**107** to methyl cinnamate. Thus, reaction of (2*S*,3*S*,5*S*,6*R*)-**107** with catalytic amounts of potassium *tert*-butoxide in CH_2_Cl_2_ at room temperature followed by the addition of methyl cinnamate, gave the cyclic α-amino-*C*-phosphinate (2*S*,3*S*,5*S*,6*R*)-**108** in 92% yield and with 70% diastereoisomeric excess (Scheme 47).

**Scheme 48:**
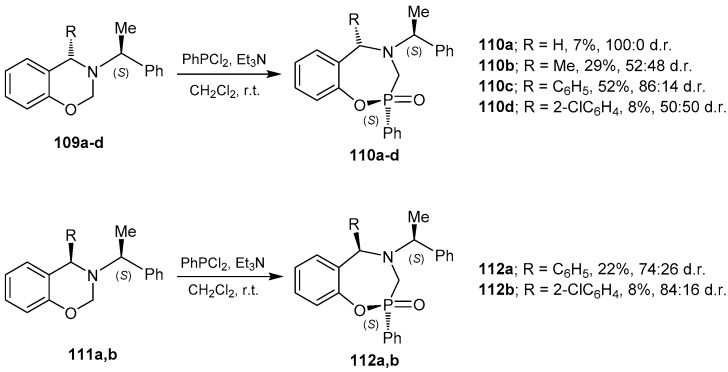
Synthesis of novel 1,4,2-oxazaphosphepines from chiral 1,3-benzoxazines.

On the other hand, Ordóñez et al. [97] carried out the stereoselective synthesis of novel 1,4,2-oxazaphosphepines from chiral 1,3-benzoxazines. For this purpose, the reaction of the chiral 1,3-benzoxazines **109a**–**d** with dichlorophenylphosphine in the presence of Et_3_N in CH_2_Cl_2_ at room temperature, gave the 1,4,2-oxazaphosphepines **110a**–**d** in 50:50 to 100:0 diastereoisomeric ratio. In a similar way, the reaction of **111a**,**b**, afforded the 1,4,2-oxazaphosphepines **112a**,**b** in 74:26 and 84:16 diastereoisomeric ratio, respectively (Scheme 48). The configuration assignment was stablished by X-ray analysis of final products.

### 3.2. 1,2-Oxaphosphacycles

**Scheme 49:**
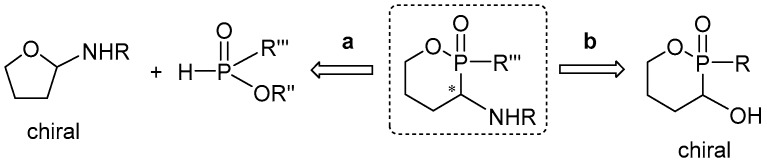
Retrosynthetic analysis of the 1,2-oxaphosphacycle scaffold.

The stereoselective synthesis of 1,2-oxaphosphacycles with an exocyclic amino group has been performed, mainly through two pathways: (a) diastereoselective nucleophilic addition-cyclization reaction from arylphosphinates and chiral cyclic hemiaminals; or (b) substitution with *N*-nucleophiles on derivatized chiral 3-hydroxy-1,2-oxaphosphinanes (Scheme 49).

**Scheme 50:**
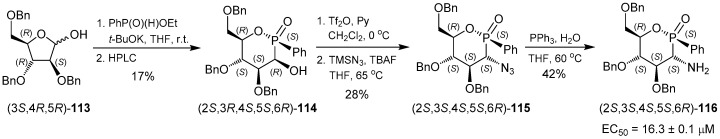
Synthesis of (2*S*,3*S*,4*S*,5*S*,6*R*)-**116** and its antiproliferative activity on glioblastoma cell line C6.

For example, the reaction of tris(benzyloxy)-d-arabinofuranose **113** with ethyl phenylphosphinate in the presence of catalytic amounts of potassium *tert*-butoxide followed by preparative reverse phase HPLC separation, led to the cyclic α-amino-*C*-phosphinate (2*S*,3*R*,4*S*,5*S*,6*R*)-**114** in 17% yield. The reaction of (2*S*,3*R*,4*S*,5*S*,6*R*)-**114** with triflic anhydride in the presence of pyridine in CH_2_Cl_2_ at 0 °C, produced the triflate, which without additional purification it was reacted with trimethylsilylazide in the presence of TBAF in THF at 65 °C, obtaining the azido derivative (2*S*,3*S*,4*S*,5*S*,6*R*)-**115** in 28% yield. Finally, reduction of azide group in the compound **115** with Ph_3_P/THF/H_2_O, furnished the glucose like oxaphosphinane (2*S*,3*S*,4*S*,5*S*,6*R*)-**116** in 42% yield, which was tested for their antiproliferative activity in the search for new therapeutic agents against glioblastoma (Scheme 50) [98].

**Scheme 51:**
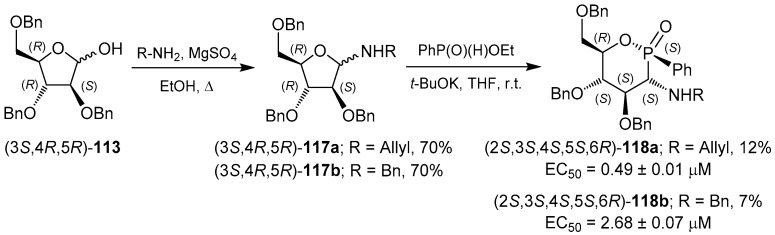
Synthesis of **118a**,**b** and its antiproliferative activities against glioblastoma cell line C6.

Additionally, the reaction of tris(benzyloxy)-d-arabinofuranose **113** with allylamine or benzylamine in the presence of MgSO_4_ in EtOH at reflux, provided the cyclic hemiaminal ethers **117a**,**b** in 70% yield, which by nucleophilic addition of ethyl phenylphosphinate and cyclization in the presence of catalytic amounts of potassium *tert*-butoxide, afforded the 3-alkylamino-1,2-oxaphosphinanes **118a**,**b** in 12% and 7% yield, respectively (Scheme 51) [98].

**Scheme 52:**
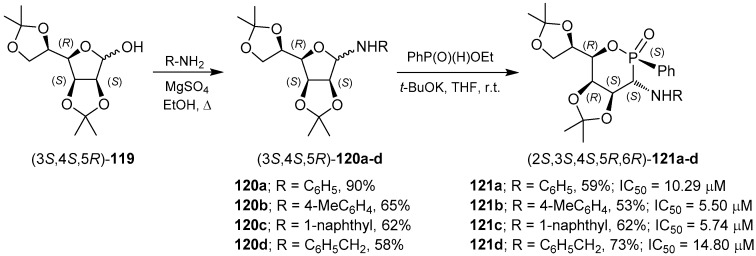
Synthesis of the 3-aryl or 3-alkylamino-1,2-oxaphosphinanes (2*S*,3*S*,4*S*,5*R*,6*R*)-**121a**–**d** and its antiproliferative activities toward glioblastoma cell line C6.

Finally, Bakalara et al. [99] carried out the synthesis of 3-amino-1,2-oxaphosphinanes as *C*-glycoside mimetics and new pharmacological agents against glioma stem cell proliferation, migration and invasion. In this respect, the condensation of the 2,3,5,6-di-*O*-isopropylidene-α-d-manno-furanose (3*S*,4*S*,5*R*)-**119** with different amines in the presence of MgSO_4_ in EtOH at reflux, afforded the cyclic hemiaminal ethers (3*S*,4*S*,5*R*)-**120a**–**d** in 58%–90% yield, which by reaction with ethyl phenylphosphinate and cyclization in the presence of catalytic amounts of potassium *tert*-butoxide, produced the 3-aryl or 3-alkylamino-1,2-oxaphosphinanes (2*S*,3*S*,4*S*,5*R*,6*R*)-**121a**–**d** in 53%–73% yield (Scheme 52).

## 4. Conclusions

In this review, we have covered recent progress in the development of stereoselective methodologies for the synthesis of both acyclic and cyclic α-amino-*C*-phosphinic acids and derivatives. As we have shown, a number of optically active α-amino-*C*-phosphinic derivatives are now accessible through different strategies; however, there is still a long way to be covered, considering the poor availability of stereoselective methods for the preparation of pure stereoisomers of phosphinic peptides, taking into account that diastereoisomers with a particular three dimensional structure are able to interact efficiently with the receptor binding site, while other configurations are discriminated. Authors believe that much effort must be made in this direction and further advances in the search and improvement of synthetic procedures, new chemical applications and biological activities of these interesting compounds will be a very rewarding task in coming years.

## Abbreviations

The following abbreviations are used in this manuscript: Ac: acetyl; ACE: angiotensin converting enzyme; APN: alanyl aminopeptidase; Ar: aryl; Boc: *t*-butyloxycarbonyl; BOP: (benzotriazol-1-yloxy)tris(dimethylamino)-phosphonium hexafluorophosphate; BPPM: (2*S*,4*S*)-*N*-butoxycarbonyl-4-diphenylphosphino-2-diphenyl-phosphino methylpyrrolidine; BSA: *N*,*O*-bis(trimethylsilyl)acetamide; BSTFA: *N*,*O*-bis(trimethylsilyl)trifluoro acetamide; Cbz: benzyloxycarbonyl; CEC: capillary electrochromatography; cod: 1,5-cyclo octadiene; d.e.: diastereomeric excess; DCC: *N*,*N'*-dicyclohexylcarbodiimide; DIPEA: diisopropyl ethylamine; DMAP: 4-dimethylaminopyridine; DMF: dimethylformamide; DNP: 2,4-dinitro phenol; e.e.: enantiomeric excess; ECE: endothelin converting enzyme; EDC: 1-ethyl-3-(3′-dimethylaminopropyl)carbodiimide; ERAP1: endoplasmic reticulum aminopeptidases 1; ERAP2: endoplasmic reticulum aminopeptidases 2; Et: ethyl; HCV NS3: hepatitis C virus nonstructural protein 3; HMDS: bis(trimethylsilyl)amine; HNC: human neutrophile collagenase; HNE: human neutrophile elastase; HOBt: 1-hydroxybenzotriazole; HPLC: high performance liquid chromatography; *i*-Bu: *iso*-butyl; IC_50_: half maximal inhibitory concentration; *i*-Pr: *iso*-propyl; LAP: leucine aminopeptidase; LDA: lithium diisopropylamide; LiTMP: lithium tetramethylpiperidide; Me: methyl; MMPs: matrix metalloproteinases; MPa: megapascal; MurD: d-glutamic acid-adding enzyme; NaHMDS: sodium hexamethyl-disilazide; *n*-Bu: *n*-butyl; NCS: *N*-chlorosuccinimide; NEP: neutral endopeptidase; nM: nanomolar; PcAm: penicillin amidase; Ph: phenyl; Phth: phthaloyl; Py: pyridine; r.t.: room temperature; RP-HPLC: reversed phase high performance liquid chromato-graphy; SDS: sodium dodecyl sulfate; *sec*-Bu: *sec*-butyl; TBAF: tetra-*n*-butyl-ammonium fluoride; *t*-Bu: *tert*-butyl; Tf: trifluoromethanesulfonate; TFA: trifluoroacetic acid; THF: tetrahydrofuran; TMEDA: tetramethylethylenediamine; TMSCl: chlorotrimethylsilane; TMSN_3_: trimethyl-silylazide; Ts: tosyl.
